# Preconditioning the immature lung with enhanced Nrf2 activity protects against oxidant-induced hypoalveolarization in mice

**DOI:** 10.1038/s41598-020-75834-8

**Published:** 2020-11-04

**Authors:** Chandra M. Tamatam, Narsa M. Reddy, Haranatha R. Potteti, Aparna Ankireddy, Patrick M. Noone, Masayuki Yamamoto, Thomas W. Kensler, Sekhar P. Reddy

**Affiliations:** 1 Department of Pediatrics, University of Illinois at Chicago, Chicago, IL 60612 USA; 2 Department of Medical Biochemistry, Tohoku University, Sendai, Japan; 3 Translational Research Program, Fred Hutchinson Cancer Research Center, Seattle, WA 98109 USA; 4 Present Address: Department of Pediatrics, Northwestern University Feinberg School of Medicine, Chicago, IL 60612 USA

**Keywords:** Paediatric research, Pathogenesis

## Abstract

Bronchopulmonary dysplasia (BPD) is a chronic disease of preterm babies with poor clinical outcomes. Nrf2 transcription factor is crucial for cytoprotective response, whereas Keap1—an endogenous inhibitor of Nrf2 signaling—dampens these protective responses. Nrf2-sufficient (wild type) newborn mice exposed to hyperoxia develop hypoalveolarization, which phenocopies human BPD, and Nrf2 deficiency worsens it. In this study, we used PND1 pups bearing bearing hypomorphic *Keap1* floxed alleles (*Keap1*^f/f^) with increased levels of Nrf2 to test the hypothesis that constitutive levels of Nrf2 in the premature lung are insufficient to mitigate hyperoxia-induced hypoalveolarization. Both wildtype and *Keap1*^f/f^ pups at PND1 were exposed to hyperoxia for 72 h and then allowed to recover at room air for two weeks (at PND18), sacrificed, and lung hypoalveolarization and inflammation assessed. Hyperoxia-induced lung hypoalveolarization was remarkably lower in *Keap1*^f/f^ pups than in wildtype counterparts (28.9% vs 2.4%, wildtype vs *Keap1*^f/f^). Likewise, *Keap1*^f/f^ pups were protected against prolonged (96 h) hyperoxia-induced hypoalveolarization. However, there were no differences in hyperoxia-induced lung inflammatory response immediately after exposure or at PND18. Lack of hypoalveolarization in *Keap1*^f/f^ pups was accompanied by increased levels of expression of antioxidant genes and GSH as assessed immediately following hyperoxia. *Keap1* knockdown resulted in upregulation of lung cell proliferation postnatally but had opposing effects following hyperoxia. Collectively, our study demonstrates that augmenting endogenous Nrf2 activation by targeting Keap1 may provide a physiological way to prevent hypoalveolarization associated with prematurity.

## Introduction

Preterm babies require hyperoxic ventilation in order to survive the neonatal period^1,2^. However, this therapy can alter lung development and contribute to bronchopulmonary dysplasia (BPD); a chronic lung disease occurring in ~ 35% of preterm survivors with a higher rate of incidence in preterm babies with a lower birth weight^3–5^. Recent clinical strategies—such as modifications in protective ventilation and/or supplementation of antenatal steroids, surfactant, and vitamin A—largely improved the survival of preterm infants, but the prevalence of BPD remains high^3–5^. Moreover, hospital readmission rates and poor health outcomes including susceptibility to recurrent pulmonary infections continue to be major clinical problems among BPD patients^6–8^. Experimental models of BPD have revealed genetic predisposition and pathologic mechanisms underlying BPD^9–11^. For example, oxidative stress (i.e., oxidant and antioxidant imbalance) and abnormal inflammatory responses leading to lung epithelial and endothelial dysfunction contribute to BPD pathogenesis^12^. However, selective antioxidant or anti-inflammatory treatments have produced limited beneficial effects in BPD patients^13^. Recently, stem cell therapies have shown promising results in animal models^14–16^, but their efficacy in preventing BPD associated with prematurity remains to be established^17^. Thus, identification of potential pharmacotherapies targeting endogenous protein(s) to prevent BPD and/or to accelerate the repair of shunted alveolar growth in preterm infants is a major unmet medical need.

The nuclear factor erythroid 2 like 2 (NFE2L2 or Nrf2) transcriptionally activates gene expression required for cytoprotection, anti-inflammation, tissue regeneration, and host defense^18^. Kelch-like ECH-associated Protein 1 (Keap1) is the endogenous negative regulator of Nrf2. It retains the latter in the cytosol and enables its proteosomal degradation by the Cul3-ubiquitin ligase system, thereby limiting Nrf2-regulated gene transcription. Stressful stimuli causing oxidative or electrophilic modifications of cysteine residues in Keap1 impede degradation of Nrf2 and foster its subsequent nuclear accumulation, leading to enhanced gene expression mediated through the antioxidant response element (ARE)^18^. We have shown that Nrf2 confers protection against infectious and non-infectious lung injury-related pathologies, including endotoxin^19^, hyperoxia^20^ and mechanical ventilation^21^. Impaired resolution of lung inflammation is observed in Nrf2-deficient mice after oxidant-induced acute lung injury^22^. Wildtype (Nrf2-sufficient) neonatal mice develop BPD-like pathogenesis (alveolar simplification or hypoalveolarization) after hyperoxic lung injury; moreover, genetic disruption of Nrf2 worsens it^23,24^. We therefore hypothesized that the magnitude of Nrf2 activation in preterm lungs is insufficient to optimally combat cellular stresses caused by prematurity and hyperoxic ventilation, leading to abnormal lung development. Herein, we demonstrated that augmentation of endogenous Nrf2 activity by knocking down *Keap1* expression prevents oxidant stress-induced lung hypoalveolarization in neonatal mice.

## Results

### Increased Nrf2 target gene expression in lungs of newborn pups with *Keap1* hypomorphism

**Figure 1:**
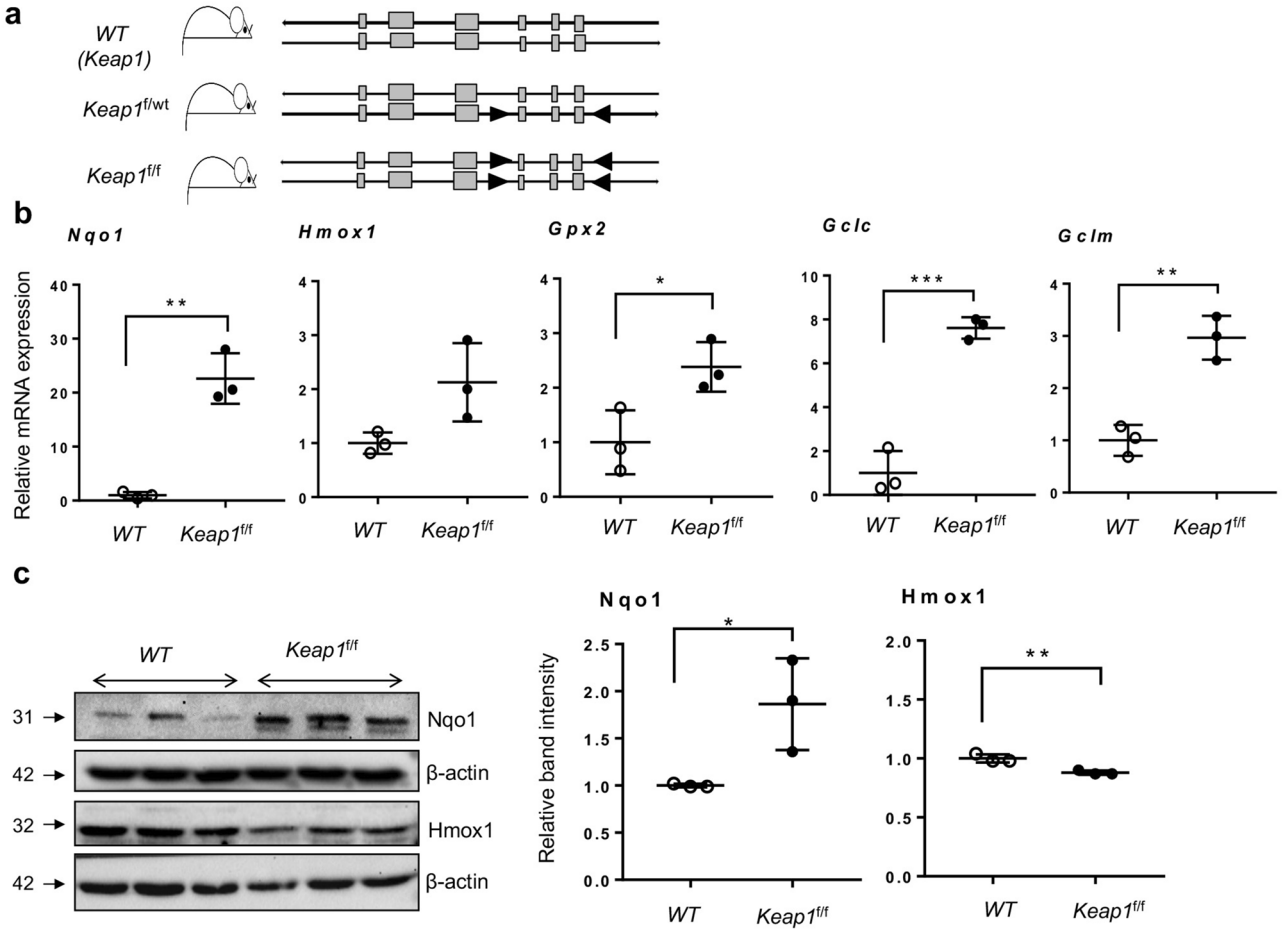
Increased expression of Nrf2 target genes in lungs of newborn *Keap1* hypomorphs. (**a**) The schema represents the *Keap1* mice bearing the heterozygous (f/wt) or homozygous (f/f) “lox P” sites (arrowheads) flanking the exons (gray boxes). The position of primers used for genotyping are indicated. (**b**) Total RNA was isolated from the lungs of 1-day-old (PND1) pups of wildtype (*WT*) and *Keap1*^f/f^ mice (n = 3), cDNA prepared, and qRT-PCR performed using gene-specific primers as indicated. (**c**) Immunoblot analyses of Nqo1 and Hmox1 in the lungs of *WT* and *Keap1*^f/f^ pups at PND1 (left). Uncropped immunoblots are presented in Supplementary Figure E1. Nqo1 and Hmox1 band intensities were quantified using β-actin band as a reference, and the values of the *WT* pups are represented as one unit (right).

Genetic disruption of *Keap1* increases both stability and nuclear accumulation of Nrf2, leading to elevated expression of Nrf2 target genes^25^. Mice bearing “floxed” alleles of *Keap1* (*Keap1*^f/f^) used in the present study are hypomorphic, wherein the insertion of “floxed” sequences within the *Keap1* intragenic region (Fig. 1A) caused knockdown of *Keap1* expression constitutively^26^. As a consequence of this knockdown, *Keap1*^f/f^ mice demonstrate increased expression of Nrf2 target genes compared to wildtype mice^26^. Lungs of one-day-old [hereafter notated as postnatal day 1 (PND1)] mice represent the lung developmental (saccular) stage of premature babies. Thus, we have analyzed Nrf2 putative target gene expression in the lungs of wildtype and *Keap1*^f/f^ pups at PND1. We chose *Nqo1*, *Hmox1*, *Gpx2,* *Gclc* and *Gclm,* as they are well characterized Nrf2 target genes and are known to play key roles in cytoprotection and cell survival in response to oxidant stress^18^. As shown in Fig. 1B, markedly increased mRNA expression of *Nqo1*, *Hmox1*, *Gpx2,* *Gclc* and *Gclm* were detectable in *Keap1*^f/f^ pups as compared to wildtype pups. Consistent with mRNA expression data, immunoblot analysis revealed increased (2.8-fold) Nqo1 levels in the lungs of *Keap1*^f/f^ pups as compared to wildtype pups (Fig. 1C). In contrast, Hmox1 expression in the *Keap1* hypomorphs was modestly lower than in wildtype pups. These results demonstrate largely increased expression of Nrf2 target genes in the newborn *Keap1* hypomorphs.

### *Keap1* hypomorphism confers protection from hyperoxia-induced lung hypoalveolarization

In order to define the effects of knockdown of *Keap1* expression (i.e., overexpression of Nrf2 target genes) on hyperoxia-induced BPD, both wildtype and *Keap1*^f/f^ pups at PND1 were exposed to room air or 95% oxygen for 72 h^27,28^. These pups were then allowed to recover at room air for two weeks (i.e., PND18), by which time neonatal mice complete their lung developmental processes including alveolarization and septae formation^29^. They were then sacrificed and the left lungs were fixed. Lung tissue sections were subjected to morphometric analysis for assessing hyperoxia-induced hypoalveolarization (also known as alveolar simplification/enlargement), a major phenotype of human BPD. We have used this exposure regime as our previous studies showed that Nrf2-deficiency worsens hypoalveolarization^23,24^. It is noteworthy that a detailed study with different levels of hyperoxia at different age of new born pups (PND1 to PND14) showed that hypoalveolarization in pups P1 to P7 exposed to 85% O_2_ followed by recovery at room air for 10 days is largely comparable to pups exposed to continuous 85% O_2_ from P1 to P14^30^.

**Figure 2:**
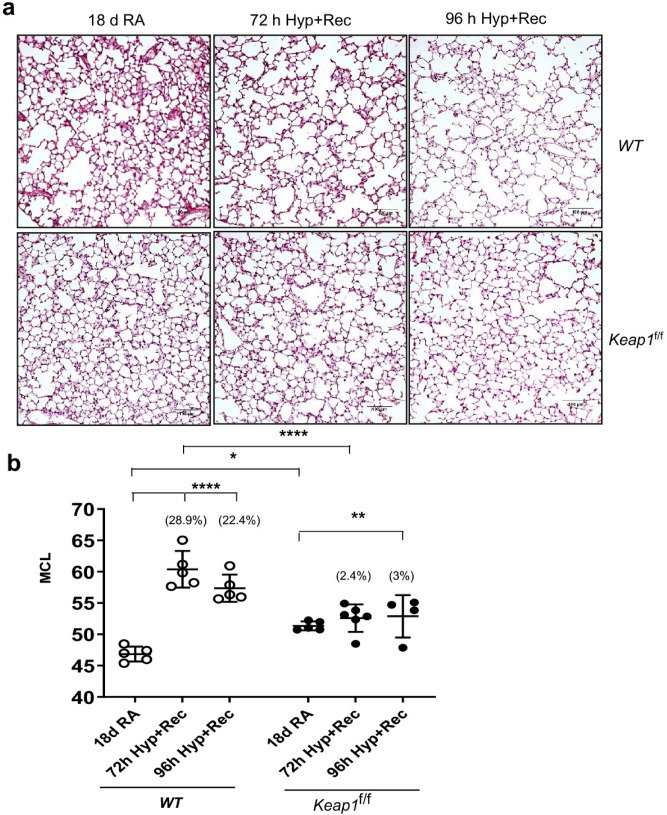
*Keap1* knockdown prevents hyperoxia-induced hypoalveolarization. Both wildtype (*WT*) and *Keap1*^f/f^ pups at PND1 were exposed to room air (RA) or hyperoxia (Hyp) for 72 h or 96 h, and pups were then allowed to recover at room air for 14 days or 13 days, respectively. Both 72 h and 96 h hyperoxia exposed mice were sacrificed at PND18, the left lung was fixed, sectioned, and stained with H&E. (**a**) Representative images of lung sections of *WT* and *Keap1*^f/f^ pups exposed to RA or Hyp at PND18 are shown (20×). Images captured with a Nikon Digital Camera are presented. Scale bar: 100 μm. (**b**) Serial H&E images of the lung Sects. (10x) were digitally captured and mean chord length (MCL) of alveolar region was analyzed by morphometry (n = 4–6).

As depicted in Fig. 2A, normal alveologenesis was largely accomplished by PND18 in both wildtype and *Keap1*^f/f^ pups exposed to room air (leftmost panels). Histopathology revealed hypoalveolarization at PND18 in wildtype neonates that were exposed to hyperoxia for 72 h at birth (top center panel), whereas *Keap1*^f/f^ counterparts lacked hypoalveolarization (bottom center panel). We next verified histological changes observed in the lung of these two genotypes by measuring mean chord length (MCL) of the alveolar region (Fig. 2B). The MCL of the wildtype neonatal mice recovered from 72 h hyperoxia increased markedly (28.9%) compared to the room air control group. In contrast, hyperoxia-induced lung hypoalveolarization was minimal in the *Keap1*^f/f^ neonatal mice (2.4%), compared to *Keap1*^f/f^ mice receiving room air. These and similar results seen with 96 h hyperoxia exposures (22.4% vs. 3%) indicated that the induction of Nrf2 signaling due to decreased *Keap1* expression in these mice provided stronger and durable protection against hyperoxia-induced hypoalveolarization compared to wildtype. Interestingly, no significant differences in the magnitude of hypoalveolarization were observed between 72 and 96 h hyperoxia-exposed wildtype mice at PND18.

### *Keap1* knockdown had no effect on hyperoxia-induced lung inflammation

**Figure 3:**
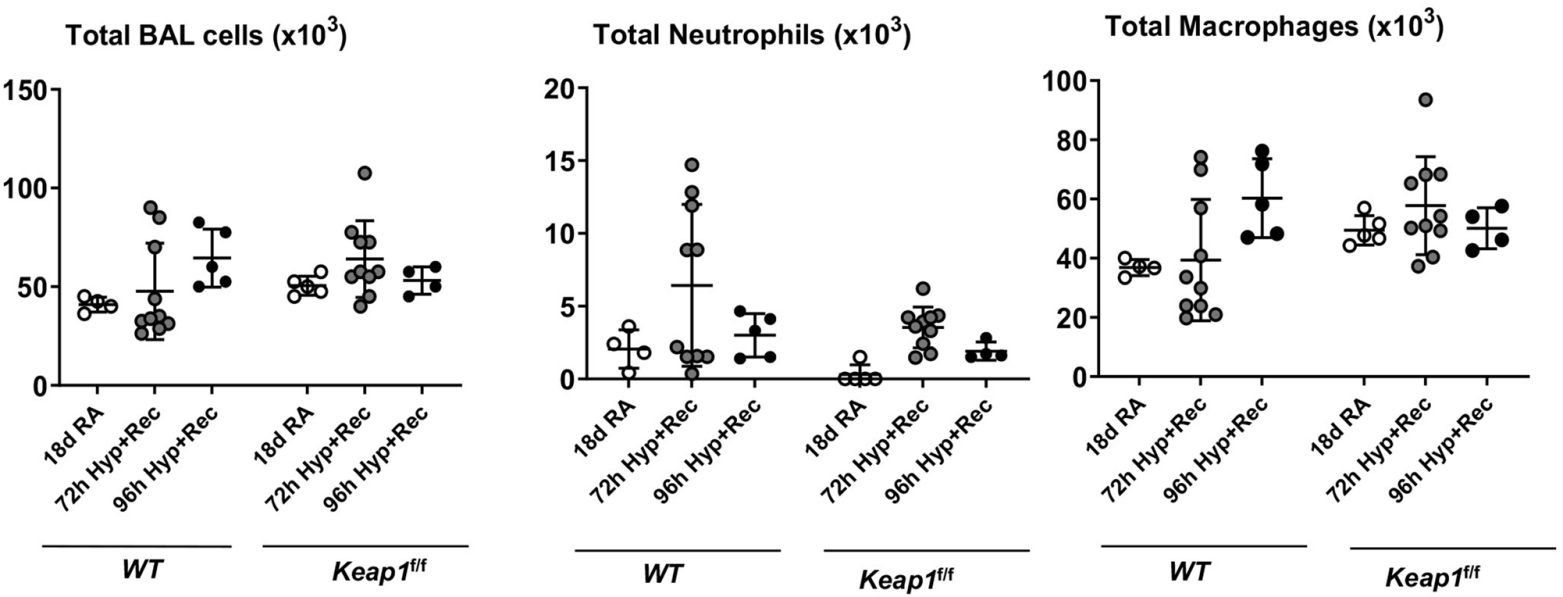
Hyperoxia-induced lung inflammation is not different between wildtype and *Keap1*^f/f^ mice. Both wildtype (WT) and *Keap1*^f/f^ pups at PND1 were exposed to 72 h or 96 h hyperoxia and then allowed to recover at room air for 14 days or 13 days, respectively. Mice at PND18 were sacrificed, the right lung was lavaged, and total cells, macrophages, and neutrophils were enumerated as detailed in methods (n = 4–10).

We next assessed lung inflammation (i.e., recruitment of leukocytes) in the BAL fluids collected from the lungs of wildtype and *Keap1*^f/f^ neonatal mice at PND18 that were exposed to room air or hyperoxia at PND1 for 72 or 96 h, and followed by recovery at room air for 14 days or 13 days, respectively. We found a modest increase in neutrophilic inflammation in the BAL fluids of hyperoxia-exposed wildtype and *Keap1*^f/f^ neonatal mice, but there were no significant differences between the two genotypes (Fig. 3).

### Hyperoxia does not affect inflammatory cytokine gene expression in newborn lungs

**Figure 4:**
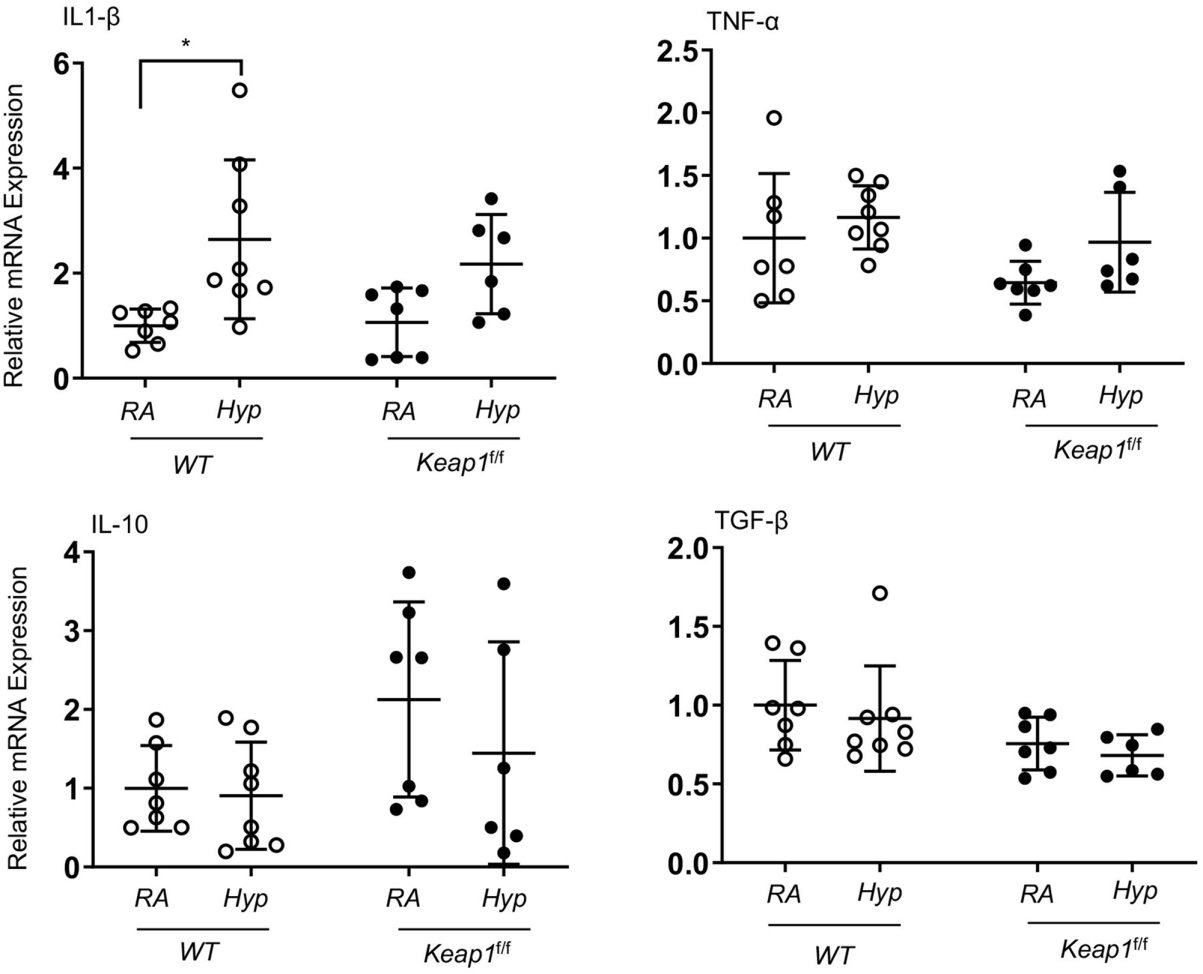
Inflammatory cytokine gene expression in wildtype and *Keap1*^f/f^ pups exposed to 72 h hyperoxia. *WT* and *Keap1*^f/f^ pups at PND1 were exposed to room air (RA) or hyperoxia (Hyp) for 72 h and immediately sacrificed (i.e., at PND4), and lungs were harvested. Total RNA was isolated, cDNA prepared, and cytokine gene expression was determined by qRT-PCR using gene-specific primers as indicated (n = 6–8).

In order to elucidate the mechanisms underlying the protective effects of *Keap1* knockdown on hyperoxia-induced alveolar simplification, both wildtype and *Keap1*^f/f^ pups were exposed to room air or hyperoxia for 72 h. RNA was isolated immediately after termination of hyperoxia exposure. The expression levels of classical pro-(IL-1β and TNF-*a*) and anti- (TGF-β1 and IL-10) inflammatory cytokines in the lungs of these two genotypes were analyzed by qRT-PCR. As shown in Fig. 4, IL-1β mRNA expression was modestly but significantly increased in the lungs of hyperoxia-exposed wildtype pups but not in their *Keap1*^f/f^ counterparts. However, TNF-*a,* TGF-β1 and IL-10 mRNA expression was not significantly altered by hyperoxia in either of the genotypes, suggesting the lack of a strong inflammatory response in the lungs of newborn pups exposed to acute hyperoxia.

### Hyperoxia-induced cytoprotective gene expression is greater in *Keap1* hypomorphic pups

**Figure 5:**
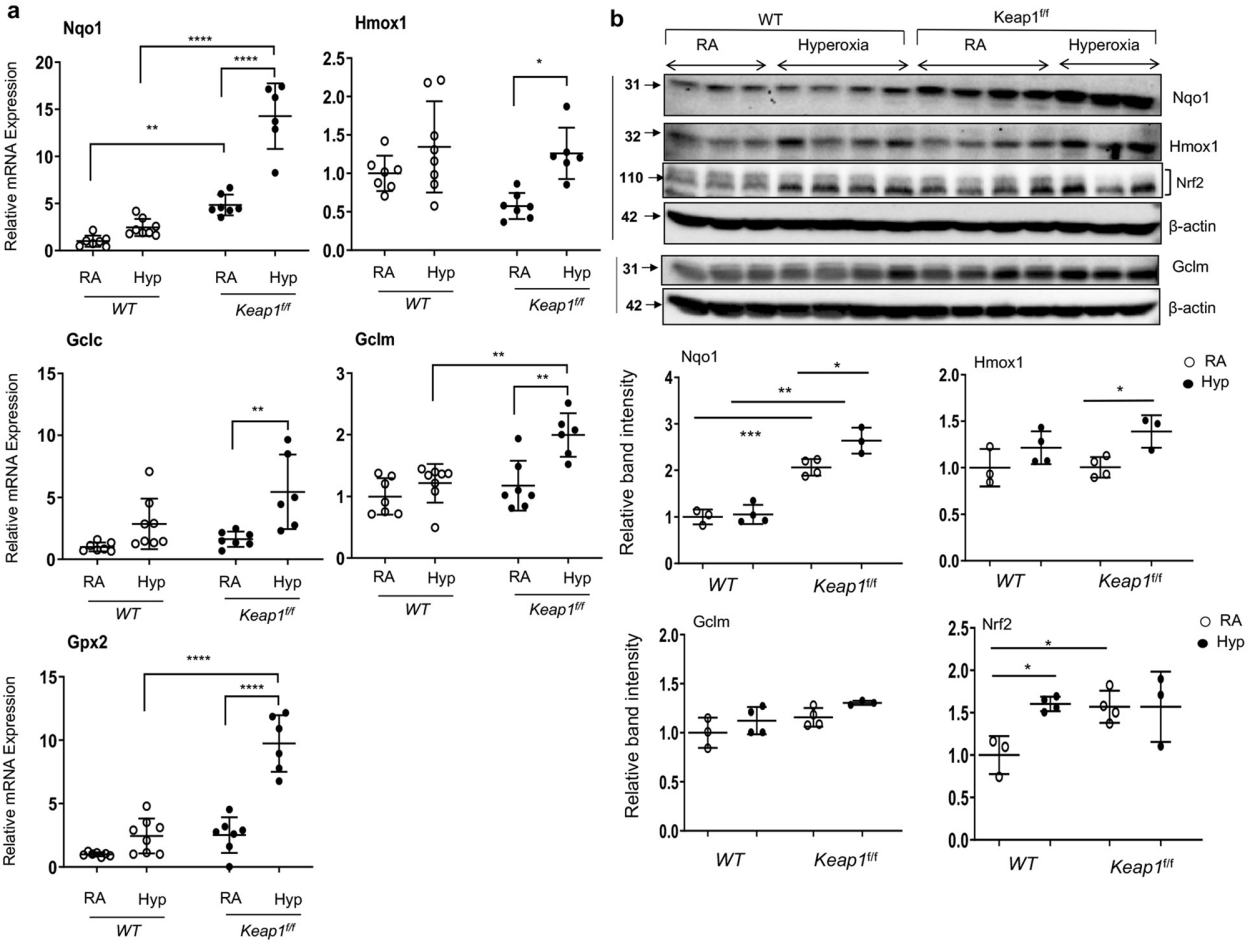
Hyperoxia-induced anti-oxidative gene expression is greater in *Keap1*^f/f^ than in *WT* pups. (**a**) Total RNA was isolated from the lungs of *WT* and *Keap1*^f/f^ neonatal pups (n = 6–8) exposed to room air (RA) or hyperoxia (Hyp) for 72 h as in Fig. 4 and subjected to qRT-PCR using gene-specific primers. (**b**) Immunoblot analyses of Nrf2, Nqo1, Hmox1 and Gclm in the lungs of *WT* and *Keap1*^f/f^ pups with and without hyperoxia exposure (n = 3–4). Uncropped immunoblots are presented in Figure E2. Protein band intensities quantified using β-actin band as a reference. The values of *WT* pups exposed to room air are represented as one unit.

**Figure 6:**
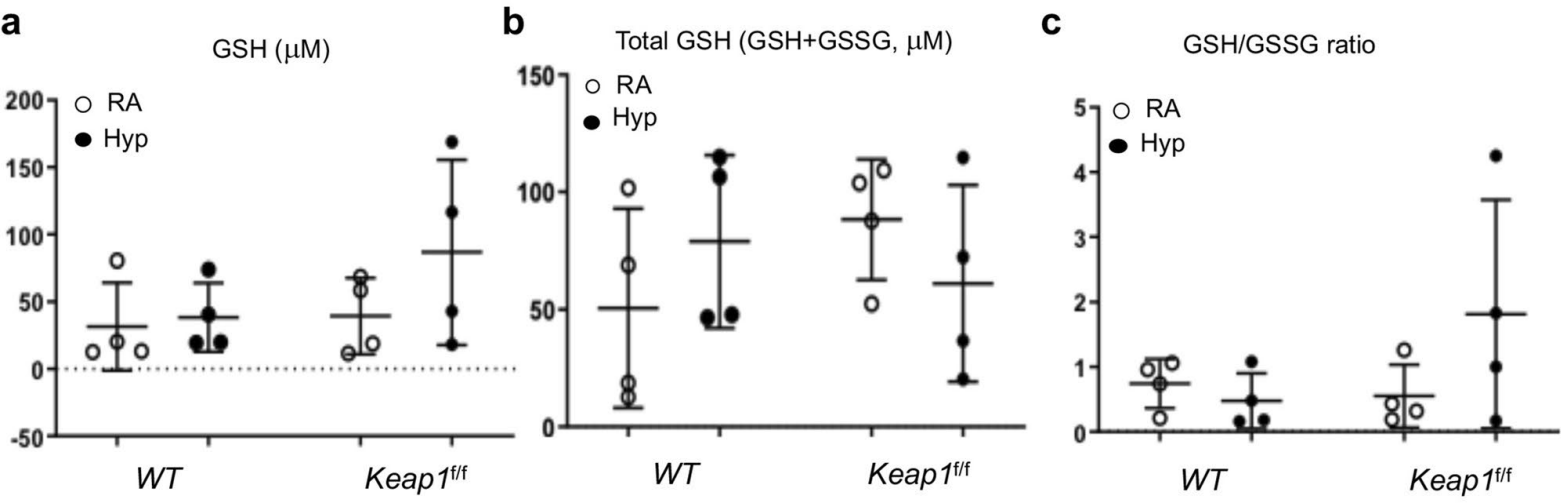
Reduced levels of oxidative stress in the lungs of *Keap1*^f/f^ pups exposed to hyperoxia. Both wildtype (*WT*) and *Keap1*^f/f^ pups (n = 4) at PND1 were exposed to room air (RA) or hyperoxia (Hyp) for 72 h, and immediately sacrificed, the right lung was frozen and used to determine levels of reduced GSH (**a**), total GSH (reduced GSH plus oxidized GSH, GSSG) (**b**), and the ratio of GSH/GSSG (**c**).

We next evaluated Nrf2-targeted antioxidant gene expression in the lungs of both wildtype and *Keap1*^f/f^ pups exposed to room air or hyperoxia for 72 h. As anticipated, basal level *Nqo1* and *Gpx2* mRNA expression was high (~ 2-threefold) in *Keap1*^f/f^ pups at PND4 compared to the wildtype pups (Fig. 5). *Nqo1*, *Gpx2*, *Gclc* and *Gclm* induction by hyperoxia was greater in *Keap1*^f/f^ pups than in their wildtype counterparts. *Hmox1* expression was significantly induced in *Keap1*^f/f^ pups exposed to hyperoxia as compared to room air controls, but its expression was not significantly induced above the basal level noted in wildtype counterparts. In agreement with mRNA expression data, immunoblot analysis revealed increased expression of Nqo1 in lungs of room air-exposed and hyperoxia-exposed *Keap1*^f/f^ pups compared to their wildtype counterparts. Both basal and inducible Gclm levels were higher in *Keap1*^f/f^ pups than in their wildtype counterparts. Hmox1 expression was modestly increased in *Keap1*^f/f^ pups exposed to hyperoxia, but its expression was not higher than in wildtype counterparts. To assess lung redox status, we determined the levels of reduced GSH (GSH) and oxidized GSH (GSSG) in wildtype and *Keap1*^f/f^ mice exposed to room air and 72 h hyperoxia (Fig. 6). We found increased levels of GSH in *Keap1*^f/f^ mice both constitutively and following hyperoxia as compared to wildtype counterparts. The GSH/GSSG ratio was lower in lungs of wildtype mice exposed to hyperoxia, but it was higher in *Keap1*^f/f^ counterparts. Taken together, these results demonstrated increased levels of hyperoxia-induced Nrf2 target gene expression and GSH and reduced levels of oxidative stress in the lungs of the *Keap1* hypomorphs.

### Keap1 hypomorphism does not affect hyperoxia-induced DNA damage response in the lung

**Figure 7:**
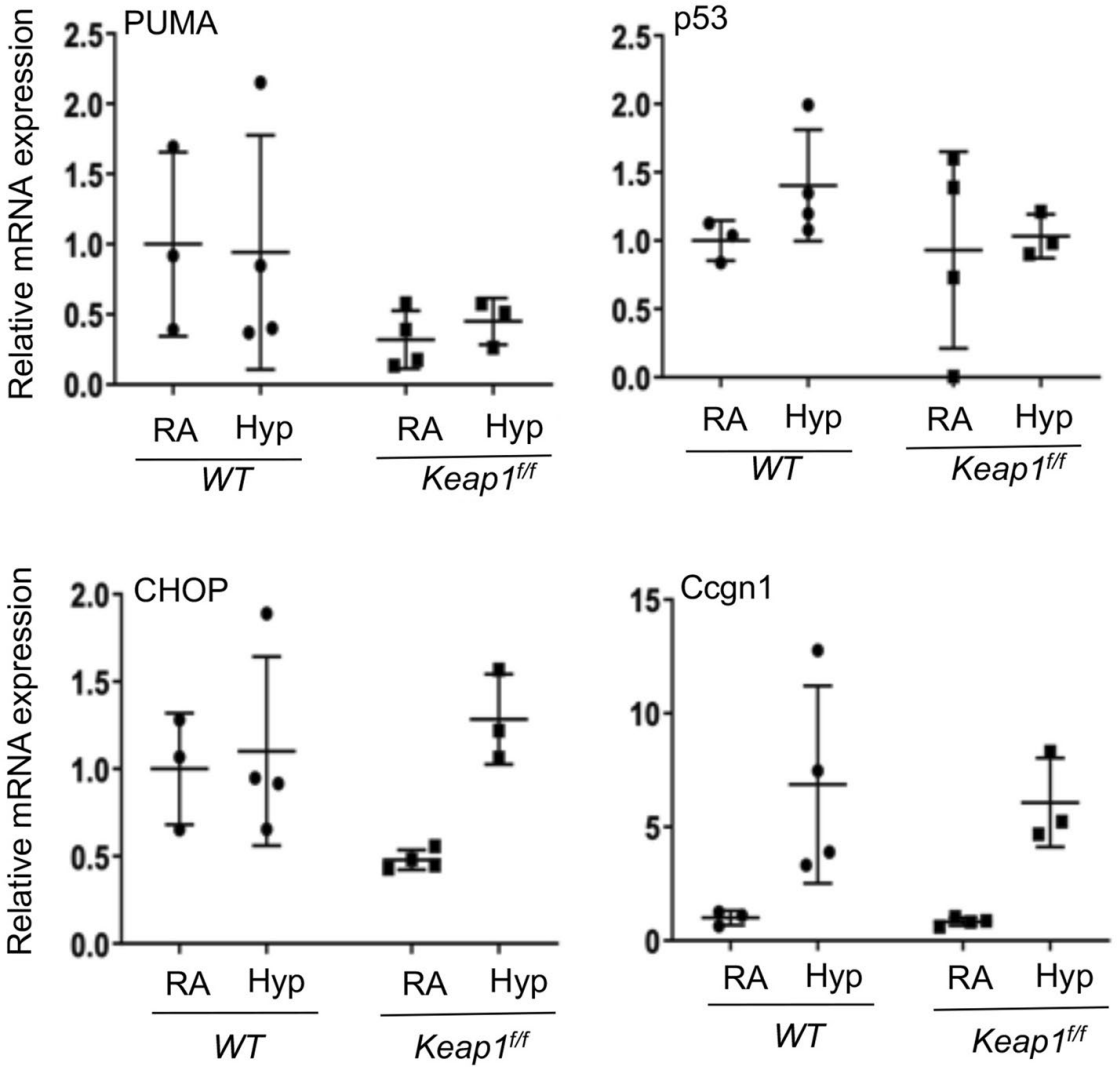
*Keap1* hypomorphism does not affect hyperoxia-induced DNA damage response pathway. As detailed above in Fig. 4, total RNA was isolated from the lungs of wildtype (*WT*) and *Keap1*^f/f^ pups (n = 3–4) exposed to room air (RA) or hyperoxia (Hyp) and subjected to qRT-PCR using gene specific primers as indicated.

To determine whether *Keap1* hypomorphism affects the activation of DNA damage response pathways induced in immature lung by hyperoxia, we quantified the expression of *p21,* *P53,* *PUMA* (p53-inducible gene), *CHOP/GADD153,* and *Ccng1* *(cyclin* *G1)* in the lungs of wildtype and *Keap1* hypomorphs exposed to hyperoxia for 72 h (Fig. 7)*.* *p53* and *PUMA* mRNA expression was not altered by hyperoxia and there was no significant difference in expression between *WT* and *Keap1*^f/f^ pups. Both *p21* and *Ccng1* mRNA expression was significantly increased by hyperoxia, but their overall induction patterns were comparable between *WT* and *Keap1* hypomorphs.

**Figure 8:**
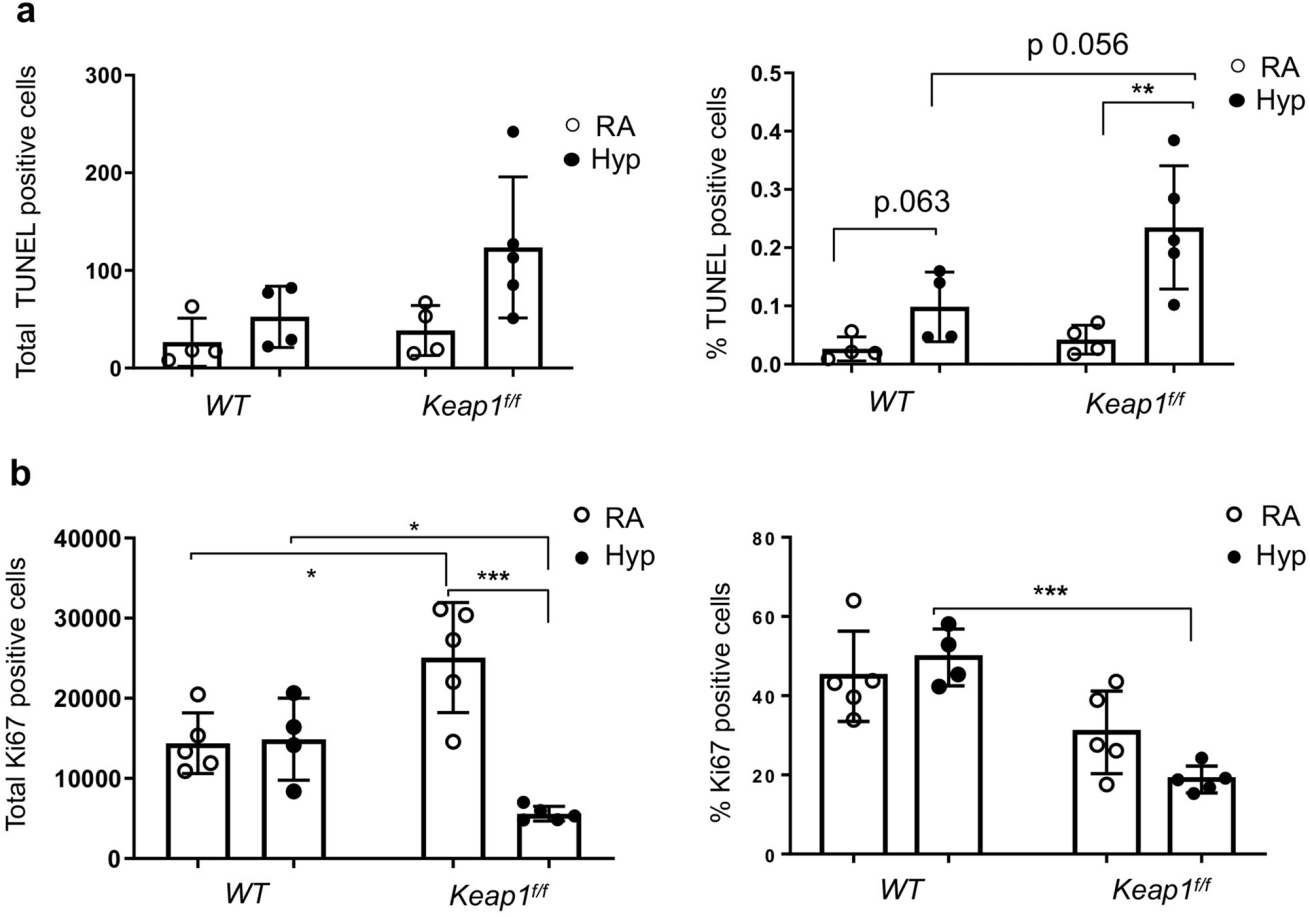
Targeting *Keap1* distinctly affects hyperoxia-induced lung cell damage and proliferation. *WT* and *Keap1*^f/f^ pups at PND1 (n = 4–5) were exposed to room air or hyperoxia for 72 h, then immediately sacrificed, the left lung was fixed, sectioned and immunostained with TUNEL or Ki-67 antibody. Images were counterstained with DAPI, captured and TUNEL-positive or Ki-67^+^ cells of the entire section were enumerated by Aperio scanning. (**a**) TUNEL-positive cells were enumerated by Aperio scanning. Left, total positive cells in the entire section; Right, % positively stained cells/total cells. (**b**) Quantification of Ki-67^+^ cells. Left, total positive cells in the entire section; Right, % positively stained cells/total cells.

To verify these findings, we next performed TUNEL staining to determine the nature of DNA damage response in vivo. Lung tissue sections of wildtype and *Keap1*^f/f^ pups exposed to room air or hyperoxia for 72 h were subjected to TUNEL staining and immunostained TUNEL^+^ nuclei (% positive and total positives) in the entire section were quantified by Aperio scanning. As shown in Fig. 8A, very few TUNEL^+^ cells were detected in the lungs of wildtype pups (26.5 positive cells or 0.026%) and *Keap1*^f/f^ hypomorphs (38.5 positive cells or 0.042%) exposed to room air. There was a modest increase in TUNEL^+^ staining in hyperoxia exposed groups, but the staining was lower in wildtype pups (52.5 positive cells or 0.098%) than in *Keap1*^f/f^ counterparts (123.6 positive cells or 0.235%). Because TUNEL staining does not necessarily correlate with DNA damage/apoptosis and also detects DNA repair and active gene transcription, we next performed active Caspase 3 staining. Caspase 3^+^ stained cells in the lungs of wildtype and *Keap1*^f/f^ pups exposed to hyperoxia were largely undetectable (data not shown). Together, these results suggested decreased levels of DNA damage and apoptosis did not protect against hyperoxia-induced hypoalveolarization in *Keap1* hypomorphs.

### Keap1 distinctly regulates lung cell proliferation both in normoxia and hyperoxia

To determine whether *Keap1* hypomorphism affects lung cell proliferation, we performed Ki-67 immunostaining in the lungs of wildtype and *Keap1*^f/f^ pups exposed to room air and 72 h hyperoxia (Fig. 8B). Ki-67^+^ cells were enumerated by Aperio scanning. The total numbers of Ki-67^+^ cells (left) and percentages of Ki-67^+^ cells (right) among total cells assessed in the lungs of wildtype pups exposed to hyperoxia were not different than in the room air control group. The total (left) or percent Ki-67^+^ cells (right) in lungs of *Keap1*^f/f^ pups exposed to hyperoxia were significantly lower than in wildtype counterparts. On the contrary, total Ki-67^+^ cells in *Keap1*^f/f^ pups exposed to room air were higher than in wildtype pups. These data suggested that Keap1 differentially regulates lung cell proliferation postnatally and in response to hyperoxia.

### Heterozygous *Keap1* pups are protected from hyperoxia-induced alveolar simplification

**Figure 9:**
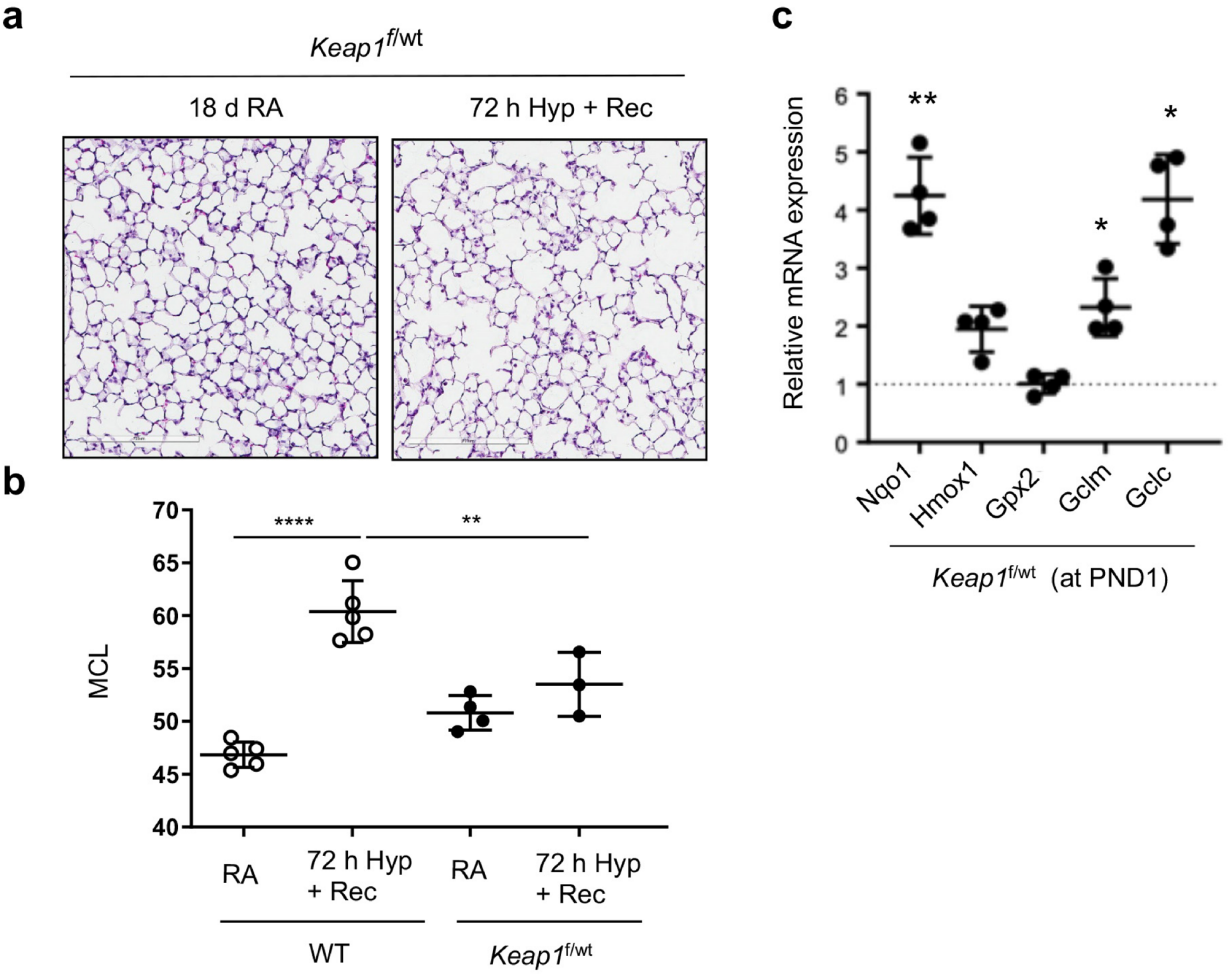
Hyperoxia-induced alveolar simplification in* Keap1*^f/*wt*^ pups. *Keap1*^f/*wt*^ pups at PND1 were exposed to room air (RA) or hyperoxia (Hyp) for 72 h, allowed to recover at room air, and sacrificed at PND18. The left lung was fixed, sectioned, and stained with H&E. (**a**) A representative image of lung sections of the *Keap1*^f/wt^ neonatal mice exposed to RA or Hyp (20x). Images captured with Aperio Image Scope are presented. Scale bar: 100 μm. (**b**) The average MCL analyzed by morphometry (n = 3). Note that the MCL of the wildtype mice used for comparison are derived from Fig. 2B. (**c**) Nrf2 target gene expression in the lungs of room-air exposed 1-day-old (PND1) *Keap1*^f/wt^ pups (n = 4). The dotted line represent values of respective genes noted in WT pups as shown in Fig. 2. *, **, compared to *WT* mice.

We next determined whether heterozygous *Keap1* hypomorphism (i.e., with a modest increase in Nrf2 target gene expression) was sufficient to confer protection against oxidant-stress induced lung alveolar simplification. To evaluate this aspect, *Keap1*^f/wt^ pups were exposed to room air or hyperoxia for 72 h, recovered at room air for two weeks, sacrificed at PND18, and the left lungs fixed. Lung tissue sections were stained with H&E and MCL was measured as detailed above. As shown in Fig. 9, both lung histopathology (panel A) and morphometry (panel B) revealed a modest increase in hyperoxia-induced alveolar simplification in *Keap1*^f/wt^ pups compared to wildtype pups. Nrf2 target gene expression analysis revealed increased levels of *Nqo1*, *Gclc,* and *Gclm,* but not *Hmox1* and *Gpx2* mRNA expression in *Keap1*^f/wt^ PND1 pups as compared to wildtype pups. Note that the levels of these gene expression in the *Keap1*^f/wt^ mice were significantly lower than in their *Keap1*^f/f^ counterparts (Fig. 1B).

## Discussion

The present study demonstrates that augmenting Nrf2-regulated gene expression through the modulation of Keap1 levels provides greater protection against hyperoxia-induced lung hypoalveolarization in neonatal mice, a major clinical manifestation of BPD associated with prematurity in humans. On the contrary, we have noted greater levels of hyperoxia-induced hypoalveolarization in *Nrf2*-null pups compared to their wild type counterparts (Tamatam and Reddy, unpublished data), consistent with previous reports^23,24^. These outcomes demonstrate an essential role for Nrf2 in dampening oxidant-induced BPD-pathogenesis. Despite significant induction of Nrf2 target gene expression, the wildtype (Nrf2-sufficient) pups develop lung alveolar impairment (i.e., hypoalveolarization) after hyperoxic injury. Taken together, these findings support our contention that constitutive levels of Nrf2 activation are insufficient to mitigate BPD development in vivo. Oxygen supplementation (hyperoxia) is required to reduce the mortality of preterm babies in the clinical setting, but this therapy is known to cause oxidative stress and impair lung development and function. While several individual antioxidant trials failed to show promising results in BPD patients^13^, the present study uncovered that targeting *Keap1* to broadly activate Nrf2-regulated gene expression may be crucial to mitigate hypoalveolarization associated with prematurity in the clinical setting.

Augmentation of constitutive Nrf2 activity by small molecules that disrupt Keap1:Nrf2 interactions was effective in conferring protection against oxidant-induced acute lung injury in adult mice^31–33^. Some of these molecules such as bardoxolone methyl, sulforaphane and naturally occurring sulforaphane-rich broccoli extract and nitro-fatty acids are in clinical trials for treatment of several lung diseases such as pulmonary hypertension, fibrosis, COPD, and asthma (clinicaltrails.gov). Newborn pups of pregnant mice treated with sulforaphane when exposed to hyperoxic stress showed reduced levels of lung inflammation and the DNA damage response pathway, but this treatment in utero could not prevent hypoalveolarization^34^. Pharmacological inhibition of thioredoxin reductase 1 activity by aurothioglucose protected pups from hyperoxia-induced alveolar simplification accompanied by Nrf2 activation and its target gene expression in C3H/HeN mice^35^. Paradoxically, this inhibitor failed to prevent hyperoxia-induced alveolar simplification in C57BL6 neonatal mice and and was associated with lack of sustained *Nqo1* and *Hmox1* activation^36^, suggesting a strain-dependent efficacy of this drug in vivo at least in mice. We found that *Keap1*^f/wt^ pups (bearing heterozygous “floxed” allele) were also protected from experimental BPD in a manner similar to *Keap1*^f/f^ mice with homozygous “floxed” alleles (Fig. 9), indicating that even modest levels of Nrf2 target gene expression may be sufficient to block the detrimental effects of supplemental oxygen on lung development. Additionally, *Keap1*^f/f^ neonatal mice are protected from hypoalveolarization induced by more prolonged hyperoxia (96 h) implying that targeting Keap1 to broadly activate Nrf2 target gene expression may provide novel means to mitigate the incidence of BPD associated with prematurity. Whether *Keap1* targeted augmentation of constitutive Nrf2 activity can reverse pre-existing hypoalveolarization in vivo warrants further studies.

Mechanistically, we found an increased expression of Nrf2 target genes (*Nqo1,* *Gpx2*, *Gclc,* and *Gclm*) in the lungs of *Keap1*^f/f^ pups exposed to hyperoxia compared with their wildtype counterparts. In the basal (constitutive) state, *Keap1*^f/f^ pups also demonstrated elevated expression levels of Nqo1 and Gpx2 but not Gclc and Gclm genes in the lungs. Inflammation contributes to hyperoxia-induced acute lung injury^37^. However, our findings revealed no striking differences in the expression level of several inflammatory mediators (e.g., TNF-α, IL-10, or TGF-β) between hyperoxia-exposed wildtype and *Keap1*^f/f^ pups. In fact, their expression in the lungs was modestly induced by hyperoxia in both genotypes, consistent with a lack of significant inflammatory cell recruitment in the lungs of both wildtype mice and *Keap1*^f/f^ neonatal mice following hyperoxic injury (Fig. 3). p53 is a key component of the DNA damage response pathway and a transcription factor that promotes cell cycle arrest and apoptosis during lung injury^38^. The activation of p53 and its target gene expression in the lung has been observed in both adult and neonatal mice following hyperoxia^38,39^. By 72 h of hyperoxia we found induction of p21 and Ccng1 gene expression but not p53 and its downstream target genes PUMA and CHOP in the lungs of both wildtype mice and Keap1 hypomorphs, and there was no difference in their mRNA expression levels between the two genotypes. This result suggests a distinct activation pattern of p53 target gene expression in lungs of newborn pups that is unaltered by *Keap1* hypomorphism. Consistent with this result, no significant difference in cell cycle regulatory gene induction was observed between wildtype and *Nrf2*-null pups exposed to hyperoxia for 72 h^34^. Lack of a drastic difference in the DNA damage response pathway activation between wildtype and *Keap1*^f/f^ neonatal pups and lack of detectable levels of active Caspase 3 staining in hyperoxic lungs coupled with the observation that *Keap1*^f/f^ pups express higher levels of antioxidant genes strongly suggests that DNA fragmentation/apoptosis may not play a larger role in mediating the protective effects of *Keap1* knockdown on hyperoxia-induced hypoalveolization.

TUNEL immunostaining studies performed in the lungs of both wildtype and *Keap1*^f/f^ pups exposed to hyperoxia revealed counterintuitive results. *Keap1*^f/f^ hypomorphs, despite being protected from hypoalveolization, showed increased level of TUNEL^+^ staining after hyperoxia exposure as compared to wildtype counterparts. While the TUNEL technique is largely used to detect DNA fragmentation/apoptosis, this method also recognizes processes related to DNA repair and active gene transcription^40,41^. Cell growth arrest for optimal DNA repair processes is crucial for restoring tissue homeostasis after hyperoxic lung injury^42^. Our studies revealed contrasting results with reduced and increased levels of Ki-67^+^ cells and TUNEL^+^ cells, respectively, in the lungs of *Keap1*^f/f^ hypomorphs exposed to hyperoxia. Thus, it is possible that growth arrest coupled with optimal repair might facilitate normal lung remodeling in *Keap1*^f/f^ pups during recovery from injury. However, further studies are warranted to substantiate this aspect.

The current study has some limitations. While our results clearly demonstrate that *Keap1* hypomorphism in mice leads to Nrf2 overexpression and mitigation of experimental BPD, the exact mechanisms through which targeting *Keap1* confers protection is unclear. For example, many different lung cell types including resident and recruited myeloid cells play an important role in normal lung development and BPD pathogenesis^12^. Thus, it is unclear whether increased Nrf2 mediated gene transcription in resident and/or recruited cells is important for conferring protection against BPD pathogenesis, which needs to be investigated using cell-type specific conditional *Keap1* knockout models. We aimed at studying specified molecular and cellular responses (DNA damage and inflammation) both immediately after hyperoxia exposure and after recovery from hyperoxic stress at 14 days (PND18), which is when neonatal pups largely accomplish the development of alveolar and septal mimicking adult lung in mice^29^. Detailed studies are warranted to further define different cell-type specific responses during recovery from hyperoxic insult (i.e., between PND4 and PND18) in wildtype and *Keap1* hypomorphic pups , which undergo abnormal remodeling (hypoalveolization) and normal lung remodeling, respectively.

In summary, our studies demonstrate that Keap1 targeted augmentation of constitutive Nrf2 activity to increase antioxidant gene expression is sufficient to mitigate oxidant-stress induced lung hypoalveolarization in vivo. While therapies are limited in preventing BPD, our current finding lays the framework for the use of targeted approaches, such as reducing Keap1 expression and/or inhibiting its interaction with Nrf2, as pharmacological ways to prevent BPD pathogenesis associated with prematurity in the clinical setting.

## Methods

### Mice and hyperoxia exposure

Mice (C57BL6:albino) bearing hypomorphic “floxed” *Keap1* alleles were used and see details elesewhere^26^. One-day-old (PND1) wildtype (WT), *Keap1*^f/wt^ (heterogyzous), and *Keap1*^f/f^ (homozygous) pups (see Fig. 1A) were exposed to hyperoxia (95% oxygen) for 72 h or 96 h. Dams were rotated every 24 h during hyperoxia exposure. Room air exposed pups were used as controls. Studies with animals were performed according to humane and eithical standards recommended by the NIH and consistent with the PHS policy on Humane Care and Use of Laboratory Animals. All animal protocols were performed in accordance guidelines approved by the animal care and use committee at the University of Illinois at Chicago. *Keap1*-K1: 5′-GCACATCCTTCATCTCTCCGCACTGGG; *Keap1*-K2: 5′-CCTCCGTGTCAACATTGGCGCGACTAG; *Keap1*-K3: 5′-TCAGCTCGATGCGGTTCACC were used to identify “floxed” (350 bp) and wildtype (250 bp) alleles ^43^.

### Morphometry analysis

Lung morphometric analysis was performed as previously detailed^44^. Briefly, mice were sacrificed and the left lung was inflated with 0.8% low melting agarose in 1.5% paraformaldehyde at 10–12 cm H_2_O pressure; this process was monitored continuously until agarose was solidified. Animal was placed at 4 °C for 30 min, the left lung was excised and kept in 1.5% paraformaldehyde for 24 h at 4 °C, washed with water and preserved in 70% ethanol. Lung was dissected into three pieces (2–3 mm thick) and embedded in paraffin, sectioned (5 μm) and stained with haematoxylin and eosin (H&E). Before morphometry, slides were coded and the whole lung images were acquired with Nikon Digital Camera DXM1200 (Nikon, Tokyo, Japan) or with an Aperio AT2® scannner (Leica) at ×10. The mean chord length (MCL) of digital JPEG images of the lung were evaluated using computer-assisted STEPanizer1 software^45^ (www.stepanizer.com) with sampling grid lines 17 μm apart, ensuring one to two chords per alveolus. Lung sections with arteries, veins, or bronchioles were excluded from the analysis.

### Assessment of lung inflammation in the bronchoalveolar lavage (BAL) fluid

The right lung was lavaged twice with 0.5 mL PBS containing 1 mM EDTA, the BAL fluid was centrifuged, and total inflammatory cells were counted. For differential counting, cells were subjected to cytospin and stained with Diff-Quick Stain Set (9990700, lot # S-630, Thermo Fisher Scientific Inc, Lombard, IL), and leukocytes were enumerated in 300 cells using the standard cytological criteria.

### Quantitative real-time PCR

RNA from lung tissue was isolated using the TRIzol reagent (Thermo Fisher). cDNA was prepared using qScript cDNA Synthesis Kit (Quanta Biosciences, Gaithersberg, MD) and SYBR green quantitative real-time PCR (qRT-PCR) assay (Thermo Fisher) was performed using gene-specific primers (Table E1, Supplemental Information). β-actin was used as control to normalize and calculate the expression levels of the target genes analyzed in the study.

### GSH/GSSG analysis

Lung tissues (20 mg) were homogenized in PBS containing 0.5% NP-40, centrifuged and 25 μl extract was used for the analysis according to recommendations provided in the GSH/GSSG ratio detection assay kit (Abcam, cat# ab138881). Both GSH (reduced GSH) and total GSH (reduced GSH + oxidized GSH) concentrations (μM) were calculated using standard curves, and GSH/GSSG ratio was determined according the protocol recommendations**.**

### Immunoblotting

Lung tissues were homogenized in RIPA buffer (R0278, Sigma, USA) with protease cocktail inhibitor (P8340, Sigma, USA). An aliquot of protein (~ 60 µg) was separated, blotted on to the PVDF membrane, and probed with Nrf2 (16396-1-AP, lot # 00043875, 1:2500 dilution), Nqo1 (11451-1-AP, lot # 00051701, 1:2500 dilution), Gclm (14241-1-AP, lot 00005573, 1:2500 dilution) (obtained from Proteintech, Chicago, IL), Hmox1 (SC-10789, lot # H1415, 1:2500 dilution, Santa Cruz Inc, Santa Cruz, CA) or β-actin (A5441, Lot # 014M4759, Sigma) antibodies. Immunoblots were developed using the HyGlo ECL kit (E2400, Denville Scientific Inc, NJ) and visualized by Bio-Rad Gel Doc system and bands were quantified using ImageJ software. β-actin band values were used to normalize and calculate relative band intensities of target genes.

### Immunohsitochemical and TUNEL analyses

Lung tissues were fixed in 2% PFA overnight, washed with 70% ethanol, embedded in paraffin and sectioned (5 µM). Tissue sections were de-paraffinized followed by antigen retrieval, blocked with 3% BSA and 1% donkey serum and then incubated antibodies. For TUNEL assays, sections were stained with terminal deoxynucleotidyl transferase (TdT) dUTP nick end labeling (TUNEL) as per the manufacturer’s guidelines (Apo Tag Peroxidase In Situ Apoptosis Detection Kit, S7100, Millipore/Sigma). DAPI was used to counter stain the nuclei. For Ki-67 staining, de-paraffinized lung sections were incubated with anti-Ki-67 antibody (ab16667, Cell Signaling Technology, MA) overnight. Sections were incubated with HRP-conjugated secondary anti-rabbit antibody and developed by DAB kit (Invitrogen) and images were scanned using the Aperio ScanScope slide scanner (Leica Biosystems, IL). TUNEL-positive and Ki-67 positive cells in the entire section were enumerated and both total and percent positive cells are presented. Caspase-3 (8G10) (9665P, Cell Signaling Technology) antibody was used to detect apoptotic cells.

### Statistical analysis

Significance was calculated using GraphPad Prism software. A student’s t-test was used for comparisions between two groups. For multiple comparisons, two-way analysis of variance with Tukey’s post-hoc test was performed. The symbols *, **, ***, and **** shown in the figures represent the *P* values equal or less than 0.05, 0.01, 0.001 and 0.0001, respectively.

## Supplementary information

Supplementary Information
