# Supplementary material for: Preconditioning the immature lung with enhanced Nrf2 activity protects against oxidant-induced hypoalveolarization in mice
Source: Sci Rep. 2020 Nov 4;10:19034. doi: 10.1038/s41598-020-75834-8 (PMC7642393; doi:10.1038/s41598-020-75834-8)
Supplement: Supplementary file 1 — Supplementary Information [file 41598_2020_75834_MOESM1_ESM.pdf]

Supplemental Information

Preconditioning the immature lung with enhanced Nrf2 activity protects against oxidant-induced hypoalveolarization in mice

Chandra M Tamatam[1], Narsa M Reddy[1#], Haranatha R Potteti[1], Aparna Ankreddy[1], Patrick M Noone[1], Masayuki Yamamoto[2], Thomas W Kensler[3], and Sekhar P Reddy[1]

Table E1 - Primers used for qRT-PCR analysis

Gene	Forward primer	Reverse primer
Nqo1	TTCTCTGGCCGATTCAGAGT	GGCTGCTTGGAGCAAAATAG
Hmox1	CACGCATATACCCGCTACCT	CCAGAGTGTTCATTCGAGCA
Gclm	TGGAGCAGCTGTATCAGTGG	CAAAGGCAGTCAAATCTGGTG
Gclc	CAATGGGAAGGAAGGGGTAT	TCAGGATGGTTTGCAATGAA
Gpx2	TCTTCCTCAGGGCTCAGTGT	TGTGAAGGCCCAGAACCTAC
IL-1β	GCCTTGGGCCTCAAAGGAAAGAATC	GGAAGACACGGATTCCATGGTGAAG
TNF-α	AGCCCCCAGTCTGTATCCTT	CTCCCTTTGCAGAACTCAGG
IL-10	CCAGGGAGATCCTTTGATGA	CATTCCCAGAGGAATTGCAT
TGF-β1	TGTGGAACTCTACCAGAAATATAGC	GAAAGCCCTGTATTCCGTCTC
PUMA	GCTGAAGGACTCATGGTGAC	CAAAGTGAAGGCGCACTG
p53	CACAGCGTGGTGGTACCTTA	TCTTCTGTACGGCGGTCTCT
CHOP	CCTAGCTTGGCTGACAGAGG	CTGCTCCTTCTCCTTCATGC
Ccng1	CTTAGTAGGCCTGTCGGATCG	GCAGTTTCTGAGAGTCAGTTG
p21	GCAGTTAGGACTCAACCGTA	ACCACCACACACACCATAGAAT
β-actin	GCAAGCAGGAGTACGATGAGT	AACGCAGCTCAGTAACAGTC

Figure E1

Whole Western Blots for Figure 1B

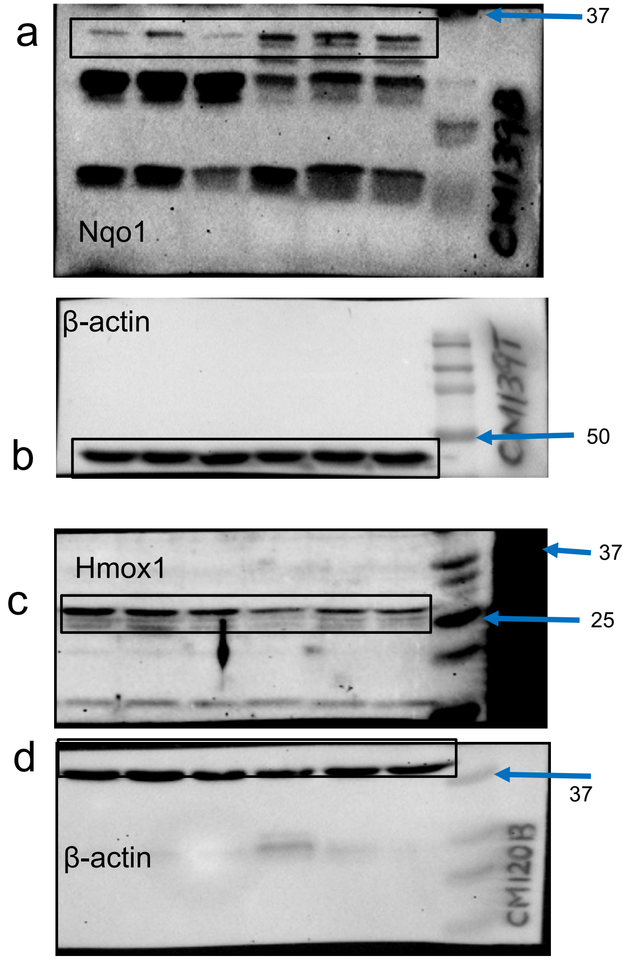

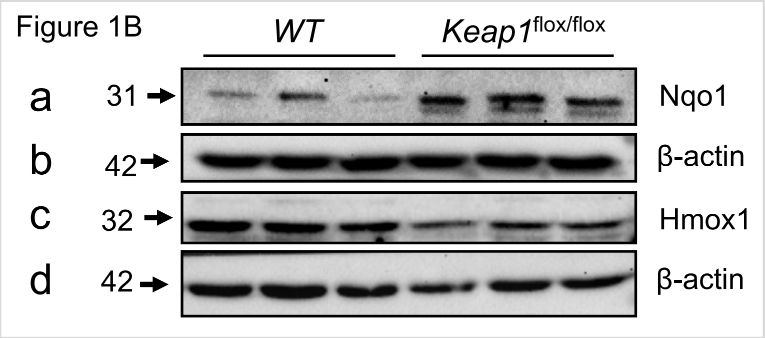

The blots of membranes (CM139 and CM120) were cut at ~40 kDa in order to probe the top (T) and bottom (B) membrane with different antibodies based on their expected sizes.

Uncropped Western Blots - Figure 5B

Figure E2

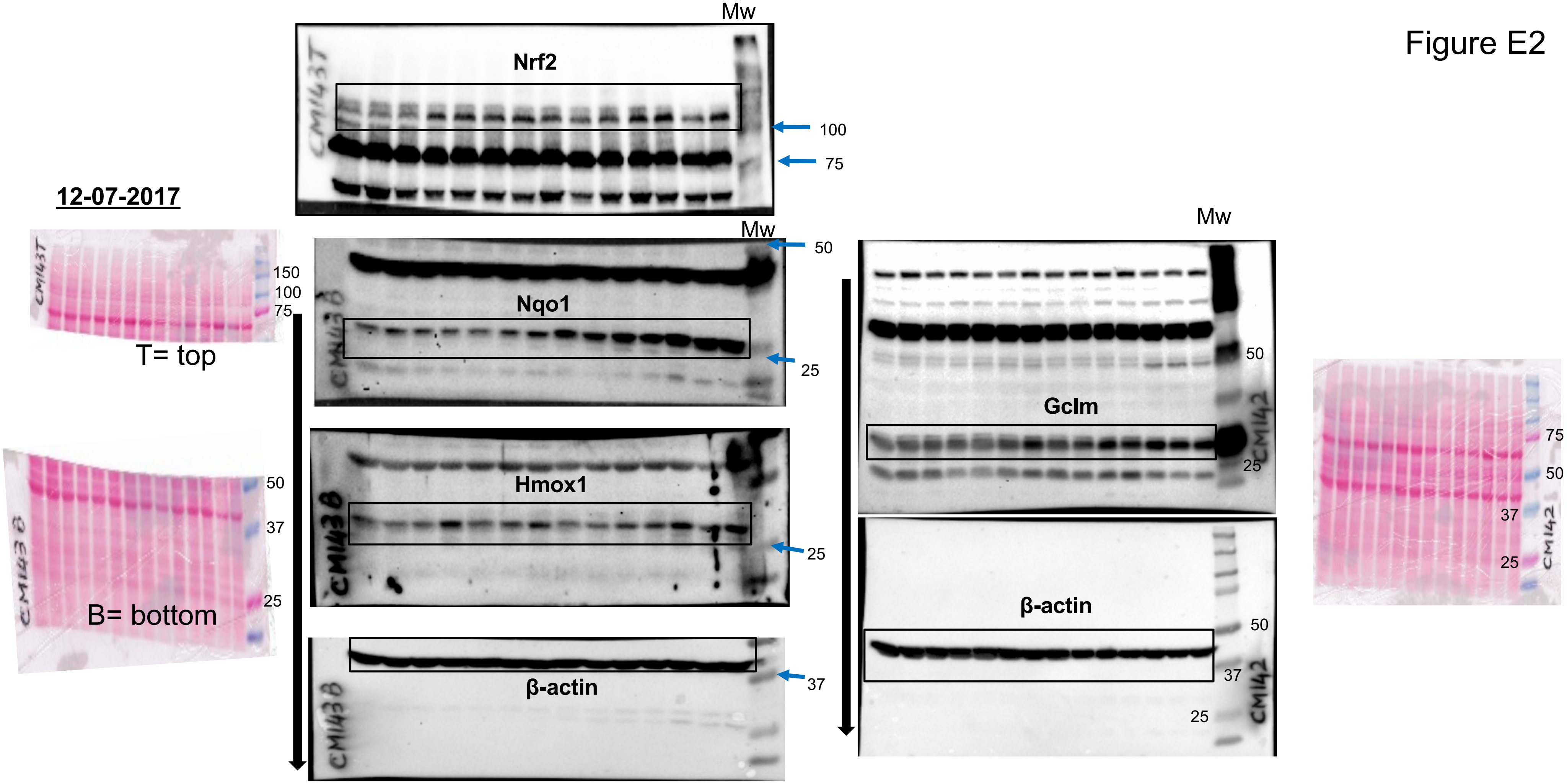

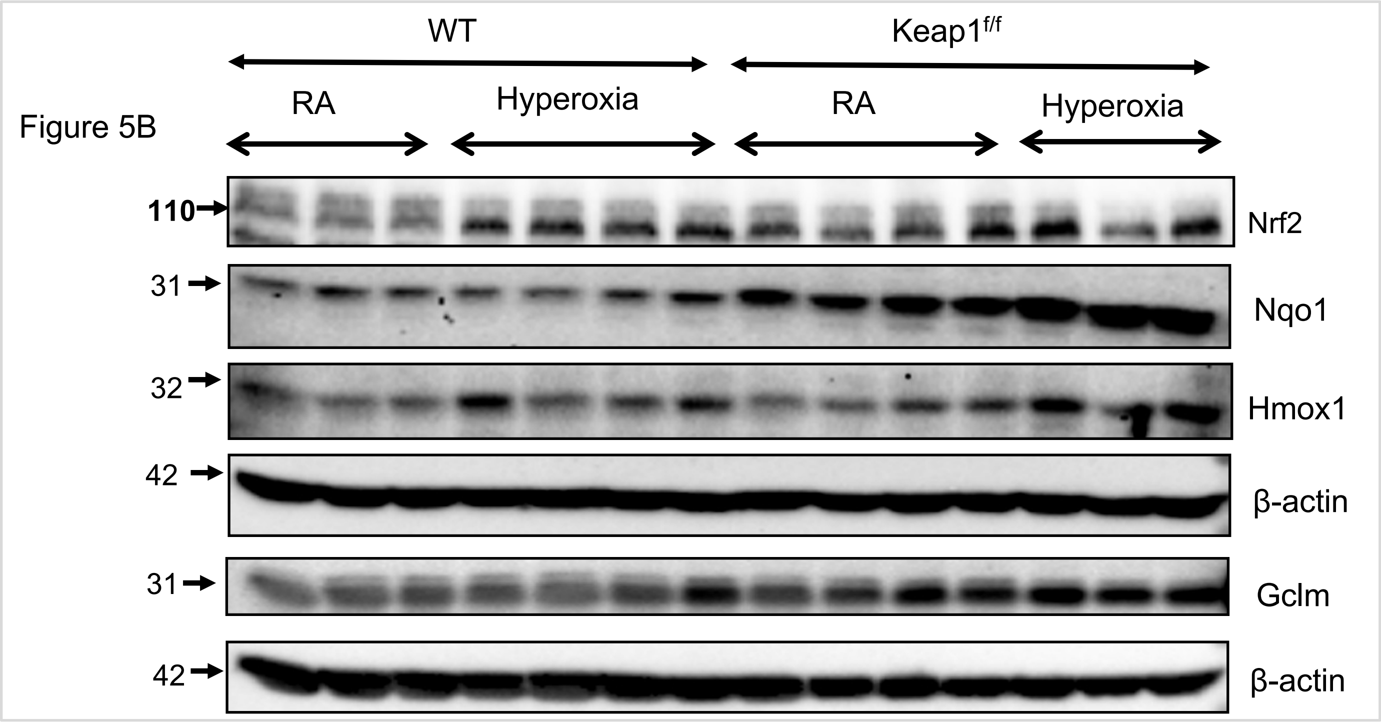

The CM143 membrane was cut at ~50 kDa in order to probe top (T) and bottom (B) membrane with different antibodies based on their molecular weight (Mw).

Filled arrows indicate the order of probing.

Mw: lane with pre-stained molecular weight markers
